# Efficacy and safety of specific treatment combined with SGLT2-i in pulmonary hypertension

**DOI:** 10.3389/fcvm.2025.1684394

**Published:** 2026-01-12

**Authors:** Chunyan Rong, Liping Zhang, Bo Li, Yin Wang, Baoguo Wang, Ming Lu, Weihua Zhang

**Affiliations:** Department of Cardiovascular Medicine, The First Hospital of Jilin University, Changchun, China

**Keywords:** pulmonary arterial hypertension, chronic thromboembolic pulmonary hypertension, specific treatment, SGLT2-i, safety

## Abstract

**Background/aims:**

Specific treatment of pulmonary hypertension (PH) can improve right heart function and exercise endurance, but many adverse reactions caused by specific drugs should not be ignored. Existing basic and clinical studies suggest that sodium-glucose cotransporter 2 inhibitors (SGLT2-i) may be useful in patients with PH and improve their prognosis to some extent. Exploring the efficacy and safety of specific treatments combined with dapagliflozin (DAPA) in patients with pulmonary arterial hypertension (PAH) and chronic thromboembolic pulmonary hypertension (CTEPH), and to provide a clinical basis for new therapeutic possibilities for pulmonary vascular disease.

**Methods:**

This study is a prospective, exploratory study that includes patients who attended the First Hospital of Jilin University from 2022 to 2024, divided into DAPA group and historical control group. The clinical data of the patients before and after treatment were compared. The primary endpoint event is defined as the improvement in 6 min walk distance (6MWD). Adverse events were defined as hemoglobin decreased, liver and kidney damage, urinary tract infection, severe hypoglycemia and hypotension, and ionic disturbances.

**Results:**

73 patients were finally included in this study, including 28 in the DAPA group and 45 in the control group, with an average age of 44.99 ± 14.14 years. After 3 months of treatment, a comparison was made between the two groups, there was no statistically significant difference in 6MWD (*P* > 0.05). Compared with before treatment, the hemoglobin (146.80 ± 24.94 vs. 139.78 ± 23.57, *P* < 0.05) in the control group decreased significantly after treatment, while the hemoglobin in the DAPA group (155.25 ± 31.30 vs. 154.04 ± 31.93, *P* > 0.05) showed no significant change.

**Conclusions:**

Studies have shown that the application of specific drugs can significantly improve the right heart function and activity endurance of patients with PAH and CTEPH. When combined with DAPA treatment, no significant additional benefits have been observed. However, DAPA treatment can alleviate the decrease in hemoglobin caused by the disease or drug reasons in patients with PH, and reduce the side effects caused by specific drugs.

## 1 Introduction

PH refers to sea level, at rest, and through right heart catheterization (RHC), the measured mean pulmonary artery pressure (mPAP) is greater than 20 mmHg (1). PH is a significant global health problem that affects all age groups, with a prevalence of approximately 1% in the global population and approximately 10% in older adults over 65 years of age (2). PH can cause changes in pulmonary vascular structure and function, leading to increased pulmonary vascular resistance (PVR) and increased pulmonary artery pressure, ultimately leading to right heart failure and even death (1). PAH and CTEPH are two PH types that can be used for specific treatment. PH specific treatment can improve the right heart function and activity endurance of patients, but many adverse reactions caused by specific treatment drugs cannot be ignored (2). SGLT2-i has been approved for heart failure with reduced ejection fraction (EFrHF), heart failure with mid-range ejection fraction (EFmrHF), and heart failure with preserved ejection fraction (EFpHF), there is effective in improving cardiovascular outcomes in patients (3, 4). Existing basic and clinical studies suggest that SGLT2-i may improve the prognosis of PH by reducing mPAP, pulmonary artery systolic and diastolic blood pressure, right ventricular systolic blood pressure, and reducing right ventricular hypertrophy and fibrosis (5–7). However, there are few relevant clinical studies, and the efficacy and safety are still unclear.

## 2 Methods

### 2.1 Purpose of the study

This study is a prospective, exploratory study to explore the efficacy and safety of specific treatment combined with DAPA in patients with class I PAH and class IV CTEPH in our center. This study protocol has been approved by the Ethics Committee of the First Hospital of Jilin University (Ethics Approval No.: 2024-1328), supported by the Jilin Provincial Health Science and Technology Capacity Improvement Project (Project No.: 2022LC092), and all enrolled patients have signed the informed consent form.

### 2.2 Research objects

**Figure 1:**
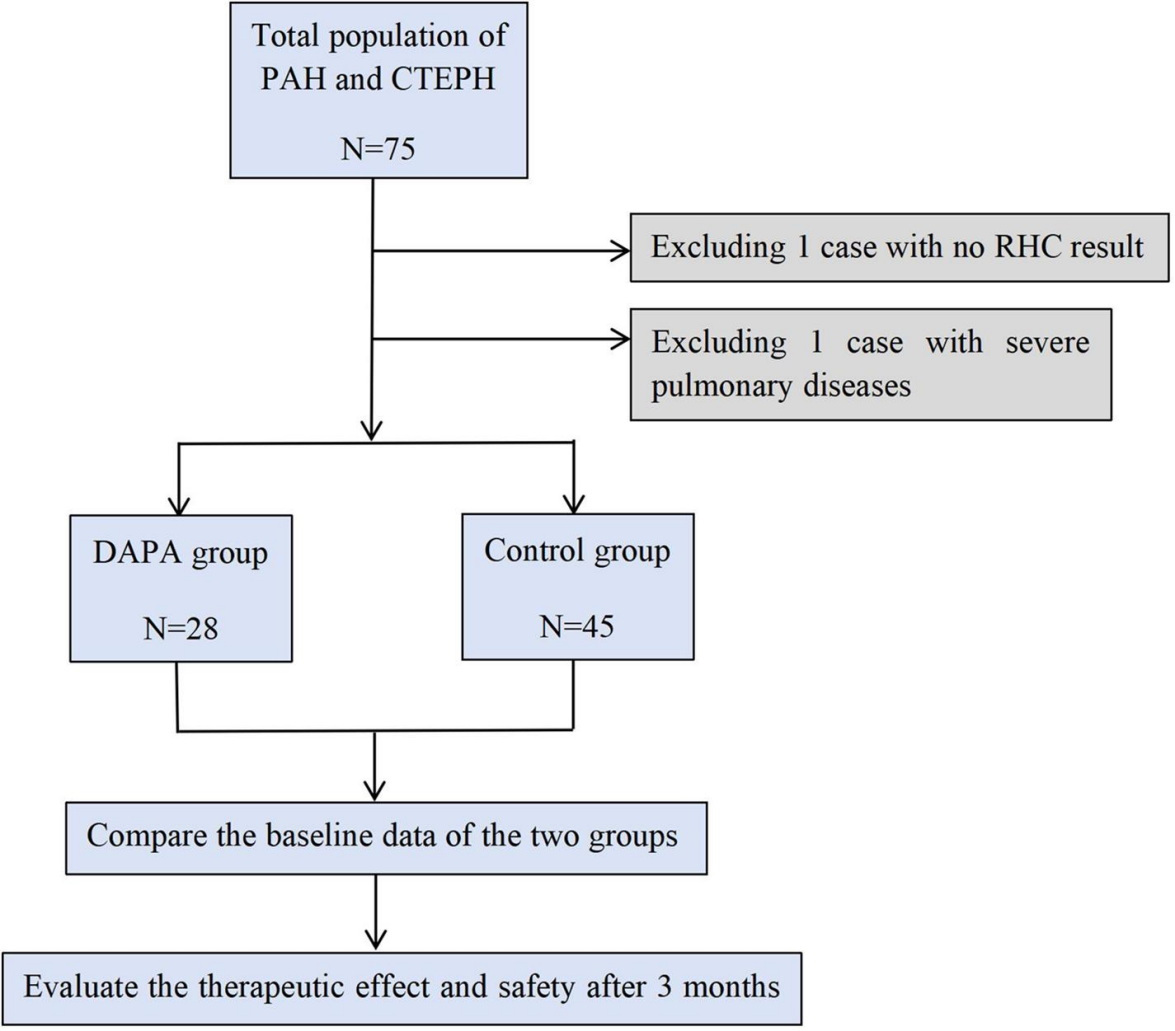
Inclusion process.

Included in the first hospital of Jilin University from January 2022 to December 2024, and diagnosed with PH by RHC; Meet the diagnostic criteria for PAH or CTEPH in the 2022 European society of cardiology/European respiratory society (ESC/ERS) guidelines for the diagnosis and treatment of pulmonary hypertension; world health organization functional class (WHO-FC) II-III Level; Voluntarily sign the informed consent form; Patients with complete clinical data. The following groups are mainly excluded: patients with PH who have not undergone RHC or who have undergone RHC and meet the criteria of II, III, V; Acute decompensated heart failure or adjustment period of less than 1 month; Inability to walk 6 min on the test; Patients with incomplete clinical data (Figure 1).

The study subjects were divided into a prospective group (DAPA group) and a historical control group (control group). Prospective group: Patients with PAH and CTEPH who attended the Department of Cardiovascular Medicine, the First Hospital of Jilin University from July 2024 to December 2024 were included and received DAPA treatment combined with specific treatment. Historical control group: Patients with PAH and CTEPH who attended the Department of Cardiovascular Medicine, the first Hospital of Jilin University from January 2022 to June 2024 were included and received only specific treatment (Figure 1).

### 2.3 Research methods

The demographic data, clinical manifestations, laboratory tests, hemodynamic parameters, electrocardiogram, echocardiography, 6-minute walk test, treatment plan and other clinical data of the patients before and after treatment were collected. Baseline initial treatment is the basic therapy for PH, including diuretics, digoxin, etc., as well as specific drug treatments, including endothelin receptor antagonist (ERA), phosphodiesterase type 5 inhibitors (PDE5i) and soluable guanylate cyclase (sGC) agonists, prostacyclin analogues and prostacyclin receptor agonists, and calcium channel blockers that are only used for patients with positive acute vascular responses. The DAPA group was treated with DAPA in combination with specific treatment (starting dose of 10 mg once a day). During the treatment, the vital signs, liver and kidney function, ion and blood glucose levels of the two groups were monitored, and the efficacy and safety were evaluated at 3 months of treatment. Patients were stratified at risk before and after treatment according to the COMPERA 2.0 fourtier risk model recommended by the 2022 ESC/ERS PH guidelines for diagnosis and treatment. The primary endpoint event was defined as the improvement of 6MWD, and the secondary endpoints were N-terminal pro-B-type natriuretic peptide (NT-proBNP), WHO-FC, risk stratification, echocardiographic indicators including right atrium diameter (RAD), right ventricle diameter (RVD), inferior vena cava diameter, tricuspid annular plane systolic excursion (TAPSE), peak velocity of tricuspid regurgitation and pressure gradient (PG) and systolic pulmonary arterial pressures (sPAP). Adverse events were defined as decreased hemoglobin, liver and kidney injury, urinary tract infection, severe hypoglycemia and hypotension, ionic disturbances.

### 2.4 Statistical analysis

SPSS29.0 software was used for statistical analysis, and the normally distributed continuous data were expressed as mean ± standard deviation ($\bar{X}\;\;\pm \;s$), and independent sample *t*-test was used for comparison between groups. The data for non-normally distributed measures were expressed as median P50 (P25, P75), and the Wilcoxon Mann–Whitney test was used for comparison between groups. Numerical data are expressed as frequencies and percentages [*n* (%)], and comparisons between groups are performed using the χ^2^ test or Fisher's exact probability method. Prism 10 was used to plot the significant difference analysis. For all statistical analyses performed, *p*-values of <0.05 were considered statistically significant.

## 3 Results

### 3.1 Baseline characteristics of the two groups before treatment

The selected PH patients were divided into 2 groups, namely the experimental group (*n* = 28) receiving DAPA intervention and the control group (*n* = 45). Among all PH patients, 20 patients (27.4%) were male and 53 patients (72.6%) were female, with a mean age of 44.99 ± 14.14 years. In terms of clinical manifestations, the most common clinical symptoms of PH patients were dyspnea in 61 patients (83.6%), and the most common signs were hyperP2 in 51 patients (69.9%) and lower extremity edema in 15 patients (20.5%). 47 patients (64.4%) with WHO-FC II and 26 patients (35.6%) with WHO-FC III were mainly included. Among all patients, 21 patients (28.8%) were at low risk, 31 patients (42.5%) were at intermediate low risk, 18 patients (24.7%) were at intermediate high risk, and 3 patients (4.1%) were at high risk (Table 1).

**Table 1:** Comparison of the results of various indicators before treatment between the DAPA group and the control group.

Index	DAPA group (*n* = 28)	Control group (*n* = 45)	*P*-value
Demographic data			
Female, *n* (%)	19 (67.9)	34 (75.6)	0.473
Age (years)	45.82 ± 14.09	44.47 ± 14.30	0.693
SBP (mmHg)	122.00 ± 21.95	119.95 ± 18.69	0.685
DBP (mmHg)	77.54 ± 15.19	77.32 ± 12.78	0.952
BMI (kg/m^2^)	22.42 ± 3.22	22.07 ± 3.76	0.706
PH type, *n* (%)			
CHD-PAH	13 (46.4)	20 (44.4)	0.904
IPAH	8 (28.6)	15 (33.3)	
CTD-PAH	3 (10.7)	6 (13.3)	
CTEPH	4 (14.3)	4 (8.9)	
Symptoms and signs, *n* (%)			
Dyspnea	21 (75.0)	40 (88.9)	0.218
Syncope	0 (0)	5 (11.1)	0.177
Hemoptysis	4 (14.3)	1 (2.2)	0.132
Cyanosis	5 (17.9)	7 (15.6)	0.796
P2 hyperactivity	22 (78.6)	29 (64.4)	0.295
Lower extremity edema	6 (21.4)	9 (20.0)	0.883
WHO-FC, *n* (%)			
II	17 (60.7)	30 (66.7)	0.606
III	11 (39.3)	15 (33.3)	
Hazard stratification, *n* (%)			
Low risk	7 (25.0)	14 (31.1)	0.702
Intermediate low risk	13 (46.4)	18 (40.0)	
Intermediate high risk	6 (21.4)	12 (26.7)	
High risk	2 (7.1)	1 (2.2)	
6MWD (m)	398.50 ± 90.27	356.16 ± 102.83	0.128
Laboratory indicators			
NT-proBNP (pg/ml)	905.00 (193.52, 1,550.00)	558.00 (183.00, 1,810.00)	0.782
Haemoglobin (g/L)	155.25 ± 31.30	146.80 ± 24.94	0.218
Blood creatinine (umol/L)	71.91 ± 20.53	65.28 ± 24.02	0.237
AST (U/L)	22.00 (17.50, 27.50)	21.60 (17.75, 28.85)	0.931
ALT (U/L)	16.20 (12.30, 25.40)	18.00 (10.70, 24.05)	0.990
LDL-C (mmol/L)	2.53 ± 0.65	2.66 ± 0.74	0.511
Blood sodium (mmol/L)	140.36 ± 2.62	139.75 ± 3.40	0.436
Blood potassium (mmol/L)	4.10 ± 0.40	3.94 ± 0.48	0.153
Fasting blood glucose (mmol/L)	5.04 ± 0.97	4.95 ± 0.77	0.712
PaO_2_ (mmHg)	73.12 ± 16.73	70.78 ± 22.25	0.706
Hemodynamic parameters			
RAP (mmHg)	10.52 ± 4.34	10.76 ± 5.88	0.856
mPAP (mmHg)	64.26 ± 25.12	57.00 ± 18.02	0.159
PAWP (mmHg)	12.29 ± 3.41	11.35 ± 3.85	0.321
PVR (WU)	14.97 (10.00, 20.00)	11.19 (6.18, 18.00)	0.117
CO (L/min)	3.89 ± 1.13	3.88 ± 1.28	0.982
CI (L/min/m^2^)	2.30 ± 0.62	2.46 ± 0.82	0.460
Electrocardiograph			
Heart rate (bpm)	82.13 ± 15.72	84.56 ± 22.74	0.671
Frontal QRS-T angle (°)	74.45 ± 43.83	74.33 ± 44.66	0.993
Right axial deviation, *n* (%)	18 (64.3)	26 (57.8)	0.581
Right ventricular hypertrophy, *n* (%)	18 (64.3)	26 (57.8)	0.581
Right bundle branch block, *n* (%)	5 (17.9)	9 (20.0)	0.821
Echocardiography			
RA left and right diameter (mm)	51.37 ± 9.94	49.90 ± 11.72	0.595
RA upper and lower diameter (mm)	56.74 ± 11.69	57.00 ± 11.97	0.930
RV anterior and posterior diameter (mm)	32.11 ± 8.08	35.49 ± 7.88	0.087
LVEF (%)	60.82 ± 5.80	61.35 ± 4.69	0.689
Inferior vena cava inner diamete (mm)	18.79 ± 4.20	17.43 ± 3.49	0.181
Inferior vena cava collapse rate >50%, *n* (%)	13 (50.0)	28 (73.7)	0.052
TAPSE (mm)	16.50 ± 3.72	15.13 ± 4.24	0.264
Peak velocity of tricuspid regurgitation (m/s)	4.31 ± 0.74	4.35 ± 0.82	0.841
Tricuspid regurgitation PG (mmHg)	76.44 ± 25.70	75.64 ± 27.90	0.904
sPAP (mmHg)	86.19 ± 29.92	87.04 ± 26.86	0.918
Treatment, *n* (%)			
ERA	27 (96.4)	39 (86.7)	0.333
PDE5i	16 (57.1)	28 (62.2)	0.666
sGC agonists	10 (35.7)	11 (24.4)	0.301
Prostacyclin analogues	2 (7.1)	3 (6.7)	1.000
Prostacyclin receptor agonists	15 (53.6)	14 (31.3)	0.057
≥ Combination of two specific drugs	26 (92.9)	37 (82.2)	0.199
Diuretics	19 (67.9)	29 (64.4)	0.765
Cardiotonic drugs	10 (35.7)	13 (28.9)	0.542

SBP, systolic blood pressure; DBP, diastolic blood pressure; BMI, body mass index; CHD, congenital heart disease; IPAH, idiopathic pulmonary arterial hypertension; CTD, connective tissue disease; AST, aspartate aminotransferase; ALT, alanine aminotransferase; LDL-C, low-density lipoprotein cholesterol; RAP, right atrial pressure; PAWP, pulmonary artery wedge pressure; CO, cardiac output; CI, cardiac index; LVEF, left ventricular ejection fraction.

Before treatment, there were no significant differences between the two groups in gender, age, SBP, DBP, BMI, PH classification, symptoms and signs, WHO-FC, risk stratification, 6MWD and other indicators (*P* > 0.05). In terms of laboratory tests, there were no significant differences between the two groups in laboratory tests such as NT-proBNP, hemoglobin, serum creatinine, AST, ALT, LDL-C, uric acid, serum sodium, serum potassium, fasting blood glucose, and PaO2 (*P* > 0.05). In terms of hemodynamics, there were no statistical differences between the two groups in RAP, mPAP, PAWP, PVR, CO, and CI (*P* > 0.05). There were no significant differences in Electrocardiograph (ECG) performance between the two groups in heart rate, frontal QRS-T angle, right axial deviation, right ventricular hypertrophy, and right bundle branch block (*P* > 0.05). In terms of echocardiography, there were no significant differences between the two groups in RA left and right diameter, RA upper and lower diameter, RV anterior and posterior diameter, LVEF, inferior vena cava inner diameter, inferior vena cava collapse rate, TAPSE, peak velocity of tricuspid regurgitation, tricuspid regurgitation PG, and sPAP (*P* > 0.05). At baseline initiation of treatment, 63 patients (86.3%) were treated with ≥2 specific drugs. There were no significant differences between the two groups in the application of ERA, PDE5i, sGC agonists, prostacyclin analogues, prostacyclin receptor agonists, ≥ combination of two specific drugs, diuretics, and cardiotonic drugs (*P* > 0.05) (Table 1).

### 3.2 Comparison of the results of various indicators between the two groups after treatment

**Figure 2:**
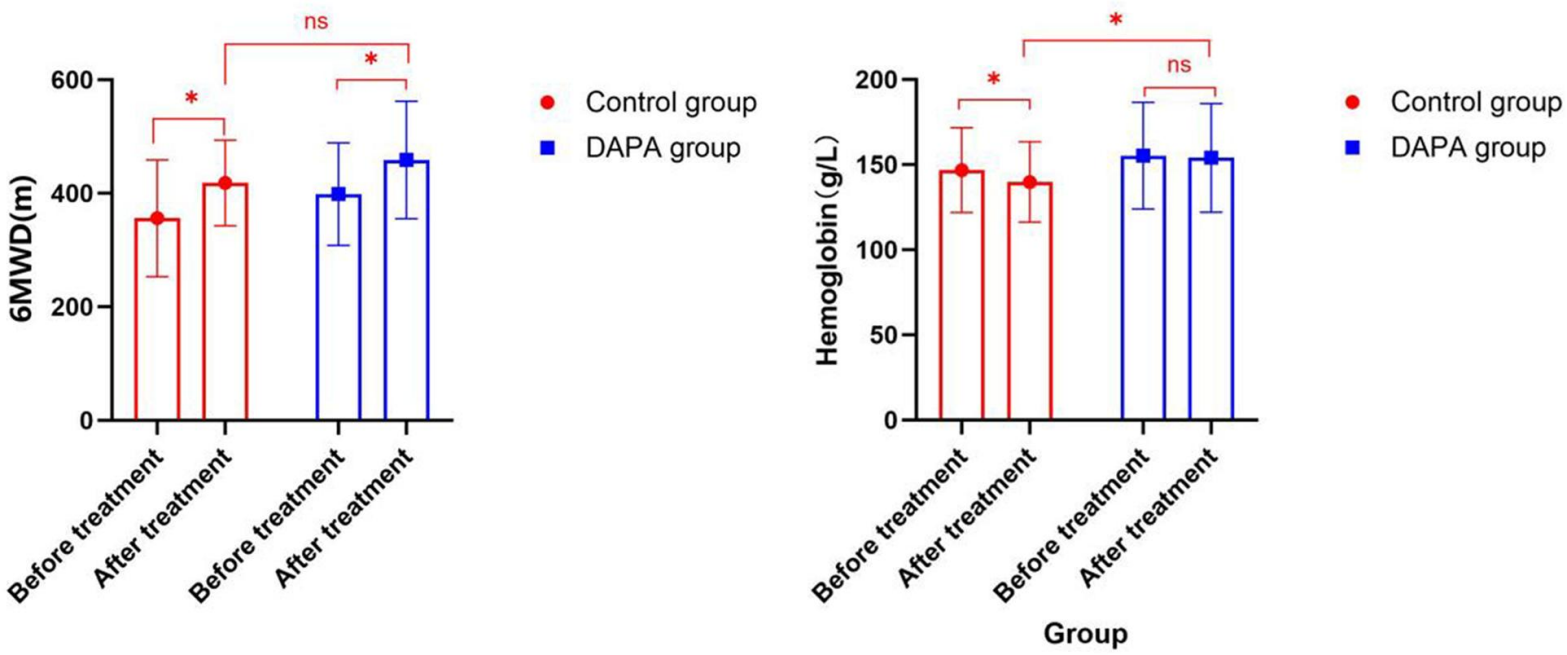
Comparison of 6MWD and hemoglobin before and after treatment in both groups within the group and between groups.

Compared with before treatment, the 6MWD of the control group (356.16 ± 102.83 vs. 418.08 ± 75.58) significantly increased, and that of the DAPA group (398.50 ± 90.27 vs. 458.60 ± 103.51) also significantly increased. However, there was no statistically significant difference between the two groups after 6MWD treatment (*P* > 0.05) (Figure 2). Compared with before treatment, the hemoglobin level of the control group (146.80 ± 24.94 vs. 139.78 ± 23.57, *P* < 0.05) significantly decreased, while that of the DAPA group (155.25 ± 31.30 vs. 154.04 ± 31.93, *P* > 0.05) showed no significant change. There was a statistically significant difference between the two groups after hemoglobin treatment (*P* < 0.05) (Figure 2). After three months of treatment, the NT-proBNP levels, RA left and right diameters, RA upper and lower diameters, RV anterior and posterior diameters, inferior vena cava diameter, peak velocity of tricuspid regurgitation and PG, sPAP, WHO-FC, and risk stratification in both groups decreased compared to those before treatment. However, there was no statistically significant difference between the groups (*P* > 0.05). There were no statistically significant differences in AST, ALT, serum creatinine, and fasting blood glucose between groups (*P* > 0.05) (Table 2).

**Table 2:** Comparison of the results of various indicators between the DAPA group and the control group after treatment.

Index	DAPA group (*n* = 28)	Control group (*n* = 45)	*P*-value
SBP (mmHg)	115.50 ± 15.52	117.62 ± 23.56	0.681
DBP (mmHg)	71.50 ± 10.72	75.84 ± 17.30	0.248
BMI (kg/m^2^)	22.01 ± 3.64	21.97 ± 3.89	0.968
WHO-FC, *n* (%)			
I	13 (46.4)	22 (48.9)	0.757
II	12 (42.9)	16 (35.6)	
III	3 (10.7)	7 (15.6)	
Hazard stratification, *n* (%)			
Low risk	14 (50.0)	21 (46.7)	0.123
Intermediate low risk	9 (32.1)	22 (48.9)	
Intermediate high risk	5 (17.9)	2 (4.4)	
High risk	0 (0)	0 (0)	
6MWD (m)	458.60 ± 103.51	418.08 ± 75.58	0.096
Laboratory indicators			
NT-proBNP (pg/ml)	723.00 (69.80, 1,250.00)	272.00 (69.30, 541.00)	0.206
Haemoglobin (g/L)	154.04 ± 31.93	139.78 ± 23.57	**0.037** *
Blood creatinine (umol/L)	72.31 ± 18.12	64.58 ± 16.84	0.074
AST (U/L)	21.00 (14.10, 27.60)	21.85 (17.10, 28.18)	0.417
ALT (U/L)	12.60 (8.80, 21.80)	15.15 (11.78, 22.40)	0.299
LDL-C (mmol/L)	2.45 ± 0.64	2.62 ± 0.69	0.354
Blood sodium (mmol/L)	139.13 ± 2.61	136.00 ± 20.90	0.444
Blood potassium (mmol/L)	4.08 ± 0.36	4.04 ± 0.37	0.635
Fasting blood glucose (mmol/L)	5.26 ± 0.90	5.13 ± 0.87	0.578
Electrocardiograph			
Heart rate (bpm)	80.83 ± 12.12	85.68 ± 14.82	0.223
Frontal QRS-T angle (°)	81.00 ± 54.73	68.08 ± 43.70	0.379
Right axial deviation, *n* (%)	11 (47.8)	12 (48.0)	0.990
Right ventricular hypertrophy, *n* (%)	12 (52.2)	12 (48.0)	0.773
Right bundle branch block, *n* (%)	2 (8.7)	7 (28.0)	0.180
Echocardiography			
RA left and right diameter (mm)	47.81 ± 9.20	47.75 ± 10.00	0.979
RA upper and lower diameter (mm)	53.67 ± 9.51	54.23 ± 10.07	0.819
RV anterior and posterior diameter (mm)	31.61 ± 7.03	33.30 ± 8.16	0.370
LVEF (%)	62.82 ± 6.42	62.60 ± 4.29	0.865
Inferior vena cava inner diamete (mm)	17.71 ± 4.66	17.06 ± 3.16	0.524
Inferior vena cava collapse rate >50%, *n* (%)	19 (73.1)	31 (81.6)	0.419
TAPSE (mm)	18.00 ± 2.38	18.54 ± 2.99	0.516
Peak velocity of tricuspid regurgitation (m/s)	4.11 ± 0.69	4.15 ± 0.76	0.821
Tricuspid regurgitation PG (mmHg)	68.18 ± 23.66	67.82 ± 23.37	0.951
sPAP (mmHg)	72.57 ± 26.48	80.50 ± 19.67	0.235

* Indicates that there is a statistically significant difference between the two groups.

### 3.3 Security analysis

Only specific treatment can cause a significant decrease in hemoglobin, but there is no significant decrease in hemoglobin in combination with DAPA. The breathing difficulties and activity endurance of 28 patients with PH who received DAPA treatment were significantly improved. During the follow-up period, their conditions and vital signs remained stable, and no significant or protocol-defined adverse events such as liver and kidney damage, urinary tract infections, severe hypoglycemia and hypotension, or electrolyte disturbances were observed in this small cohort.

## 4 Discussion

This study is a prospective, exploratory study that includes patients who attended the First Hospital of Jilin University from 2022 to 2024. The findings can be summarized as follows: Effectiveness studies have found that the application of specific drugs can significantly improve right heart function and activity tolerance in patients with PAH and CTEPH. On this basis, combined with DAPA treatment, there was no significant further improvement in right heart function and activity tolerance. Safety studies have found that DAPA treatment can improve the decrease in hemoglobin caused by disease causes or drug causes in PH patients, and reduce the side effects caused by specific drugs.

SGLT2-i has been approved for HFrEF, HFmrEF, HFpEF, and is effective in improving cardiovascular and renal outcomes in patients (3, 4). The results of basic experimental studies suggest that SGLT2-i may improve the prognosis of PH in PH rats by lowering systemic blood pressure, reducing mPAP, RVSP, reducing right ventricular hypertrophy and fibrosis, and alleviating pulmonary artery myositis in rats (8–12). Some clinical studies suggest that SGLT2-i may reduce mPAP, pulmonary artery systolic and diastolic blood pressure, and right ventricular systolic blood pressure in patients (13–15). Based on the above research background, this study took PAH and CTEPH patients as the research object, and clinically intervened with dapagliflozin to explore the efficacy and safety of SGLT2-i in PAH and CTEPH.

This study found that after 3 months of specific treatment, regardless of whether or not combined with DAPA treatment, the 6MWD of patients was prolonged compared with before treatment, and the NT-proBNP, RAD, RVD, inferior vena cava diameter, peak velocity of tricuspid regurgitation and PG, sPAP, WHO-FC, and risk stratification were all decreased compared with before treatment, and TAPSE increased compared with before treatment, but there was no statistical difference between the two groups after treatment (*P* > 0.05). On the basis of specific treatment, combined with DAPA treatment, no significant additional benefits have been observed. Previous research results have shown that ERA, PDE5i, sGC agonists, prostacyclin analogues and prostacyclin receptor agonists can improve 6MWD, mPAP, PVR, NT-proBNP concentration, clinical deterioration time and WHO-FC to varying degrees in the treatment of PAH (16, 17). For patients with CTEPH, although pulmonary endarterectomy remains the preferred treatment option for the majority of CTEPH patients, approximately 40% of the patients in the international CTEPH registry are considered ineligible for surgery. A large number of small-scale studies and three large-scale randomized controlled trials have shown that specific drug treatments can bring varying degrees of improvement to patients who are not eligible for surgery in terms of WHO-FC, 6MWD, and PVR (18–20). Tang et al.'s study demonstrated that DAPA reduced right ventricular systolic pressure and pulmonary vascular remodeling in the PAH rat model. However, in PAH rats, the combined treatment of DAPA and sildenafil was not more effective than the single treatment with sildenafil, which is similar to our research results (8).

This study found that after 3 months of treatment, the hemoglobin level in the control group decreased significantly (146.80 ± 24.94 vs. 139.78 ± 23.57, *P* < 0.05), while there was no significant change in the hemoglobin level in the DAPA group (155.25 ± 31.30 vs. 154.04 ± 31.93, *P* > 0.05). There was a statistically significant difference between the two groups after hemoglobin treatment (*P* < 0.05). There were no statistically significant differences in blood pressure, AST, ALT, serum creatinine, serum potassium, serum sodium, and fasting blood glucose between the two groups (*P* > 0.05). The assessment of the PH cohort indicates that the frequency of iron deficiency and/or anemia in PH patients is very high. Approximately 40%–60% of PH patients exhibit iron deficiency, and one-third of all PH patients suffer from anemia. Both iron deficiency and anemia significantly affect the morbidity and mortality of PH patients (21, 22). Previous studies have shown that treatment with ERA and sGC agonists for PH may lead to a decrease in hemoglobin levels (23, 24). The reason for ERA's reduction in hemoglobin is not clear, but it may be partly related to the dilution effect caused by fluid retention, and partly related to the blocking of endothelin B receptors in the glomeruli and the decrease in erythropoietin (25, 26). Levosibax usually causes mild anemia. The exact mechanism remains to be determined. Some experts suggest that this anemia might be caused by the vasodilatory effect (27).

SGLT2-i is a hypoglycemic drug that has been proven to be beneficial for the cardiovascular and renal systems of patients with type 2 diabetes mellitus (T2DM), HF or chronic kidney disease (CKD). Due to the involvement of multiple factors, the treatment with SGLT2-i may be associated with an increase in hemoglobin level of 0.5–0.7 g/dl (28). In a very short period of time, the change in hemoglobin/hematocrit was initially attributed to blood concentration, as it has a diuretic effect and reduces plasma volume (29). If used for a long period, SGLT2-i can increase hemoglobin levels by stimulating the production of endogenous erythropoietin and by activating the hypoxia-inducible factor pathway to improve iron kinetics (28). SGLT2-i can directly stimulate the increase of erythropoietin (EPO) concentration by inhibiting sodium reabsorption in the proximal tubules of the kidney, reducing the activity of Na^+^/K^+^-ATPase, decreasing the oxygen consumption of tubular cells, reversing the “relative hypoxia of renal tissue”, and restoring the EPO secretion function of peritubular fibroblasts (the main cells for EPO synthesis) (30). Furthermore, SGLT2-i can indirectly stimulate erythropoiesis by improving renal medullary oxygenation and enhancing bone marrow sensitivity to EPO, for instance, by mitigating the inhibitory effects of inflammation on erythroid progenitor cells in the context of bone marrow inflammation. This mechanism may compensate for the lack of a significant increase in EPO concentration and ultimately lead to an elevation in red blood cell production (30, 31). Iron is the core raw material for red blood cell production. SGLT2-i regulates key molecules involved in iron metabolism, thereby improving iron absorption, mobilization and utilization, providing a raw material guarantee for red blood cell production. The study found that after SGLT2-i treatment, there was a significant decrease in hepcidin and ferritin levels, an increase in erythroid ironoprotein, transferrin/total iron binding capacity and soluble transferrin receptor, and a decrease in transferrin saturation (28). Based on the effect of SGLT2-i on hemoglobin, in this study, the decrease in hemoglobin levels in the experimental group was lower than that in the control group, indicating that DAPA treatment can alleviate the decline in hemoglobin caused by the disease or drug factors in patients with PH, reduce the side effects caused by specific drugs, and improve the long-term prognosis of patients. Similar to previous studies, with the combined DAPA treatment, no significant or protocol-defined adverse events such as liver and kidney damage, urinary tract infections, severe hypoglycemia and hypotension, or electrolyte disturbances were observed in this small cohort. The clinical application was safe and effective (32, 33).

This study has the following limitations: It adopts a historical control method, with a small sample size, lacks long-term follow-up data, and may have time bias, unadjusted confounding factors, etc. Although the measurable core covariates were adjusted through statistical models, they may still have some impact on the final results. Further studies are needed to explore more comprehensive strategies for controlling confounding factors. The main population included in this study were patients with pulmonary hypertension, with a small number of patients, high disease heterogeneity, and scattered distribution. To a certain extent, this limited the implementation of randomization. In the future, we will expand the sample size based on this study, conduct multi-center randomized controlled studies as much as possible, and carry out long-term follow-ups.

## 5 Conclusion

This study was a prospective, single-center, exploratory research. The results further confirmed that for patients with PH, the application of specific drugs can significantly improve the right heart function and activity endurance of patients with PAH and CTEPH. Based on the clinical efficacy study of SGLT2-i, it was found that the combination of specific drugs with DAPA treatment did not show significant additional benefits, but it provided solid empirical evidence for clinical practice and subsequent research. The safety study found that DAPA treatment can improve the decline in hemoglobin levels in patients with PH caused by the disease or the medication, and alleviate the side effects caused by specific drugs. This study provides preliminary clinical references for the PH treatment strategy and lays the foundation for future prospective, multi-center, and large-sample randomized controlled studies.

## Data Availability

The original contributions presented in the study are included in the article/supplementary material, further inquiries can be directed to the corresponding author/s.
